# Dengue in peri-urban Pak-Ngum district, Vientiane capital of Laos: a community survey on knowledge, attitudes and practices

**DOI:** 10.1186/1471-2458-13-434

**Published:** 2013-05-03

**Authors:** Mayfong Mayxay, Wanyuan Cui, Sounthone Thammavong, Khamphong Khensakhou, Viengnakhone Vongxay, Latdaphone Inthasoum, Vanphanom Sychareun, Gregory Armstrong

**Affiliations:** 1 Faculty of Postgraduate Studies, University of Health Sciences, Vientiane, Lao PDR; 2 Lao-Oxford-Mahosot Hospital-Wellcome Trust Research Unit (LOMWRU), Mahosot Hospital, Vientiane, Lao PDR; 3 Centre for Clinical Vaccinology and Tropical Medicine, Churchill Hospital, University of Oxford, Oxford, UK; 4 Nossal Institute for Global Health, The University of Melbourne, Melbourne, Australia; 5 Pak-Ngum District Health Office, Vientiane Capital, Lao PDR

**Keywords:** Dengue, Knowledge, Attitude, Practice, Vientiane, Laos

## Abstract

**Background:**

Dengue remains an important cause of morbidity in Laos. Good knowledge, attitudes and practices (KAP) among the public regarding dengue prevention are required for the success of disease control. Very little is known about dengue KAP among the Lao general population.

**Methods:**

This was a KAP household survey on dengue conducted in a peri-urban Pak-Ngum district of Vientiane capital, Laos. A two-stage cluster sampling method was used to select a sample of participants to represent the general community. Participants from 231 households were surveyed using an interviewer-administered questionnaire.

**Results:**

Although 97% of the participants heard of dengue, there was a lack of depth of knowledge on dengue: 33% of them did not know that malaria and dengue were different diseases, 32% incorrectly believed that *Aedes* mosquito transmits malaria, 36% could not correctly report that *Aedes* mosquitoes bite most frequently at sunrise and sunset; and < 10% of them recognized that indoor water containers could be *Aedes* mosquito breeding sites. Attitude levels were moderately good with a high proportion (96%) of participants recognizing that dengue was a severe yet preventable disease. Self reported prevention methods were quite high yet observation of the participants’ yards showed use of prevention methods to be only moderate. The majority (93%) of the interviewees did not believe that they had enough information on dengue. There was an association between good knowledge and better practices, but good knowledge was associated with worse attitudes.

**Conclusions:**

There is a lack of depth of knowledge regarding dengue in Pak-Ngum community and observation methods revealed that more needs to be done by community members themselves to prevent the spread of *Aedes* mosquitoes.

## Background

Dengue - an *Aedes* mosquito-borne, viral and preventable disease – remains an important public health problem in the tropical and subtropical world [1,2]. It is estimated that ~2.5 billion people globally are at risk of dengue infection, of which 52% reside in Southeast Asia [3]. Annually, there are approximately 50 to 100 million cases of dengue infection with 500,000 severe cases requiring hospitalization which result in approximately 24,000 deaths, mainly among children [2].

In Lao PDR (Laos), dengue is still an important cause of morbidity particularly in urban areas. The disease is endemic in nine out of seventeen provinces and 3.9 million residents are presently at risk of infection [4]. Over the past decade, the burden of dengue has increased dramatically and in 2006 it was ranked one of the top ten causes of death in Laos [5]. It is observed that dengue epidemics occur cyclically, every few years [6,7]. The first reported dengue outbreak in Lao PDR was in the capital of Vientiane in 1983, where there were 1,759 cases of dengue hemorrhagic fever or DHF [8]. Since then, there have been multiple reports of outbreaks, which are now no longer restricted to just the capital. The largest of these outbreaks was in 1987, where there were 9,699 cases and 295 deaths, mostly in children under fifteen years of age [8]. In 2009, 7,835 dengue cases and 19 deaths were reported to the national surveillance system (facility-based), which increased to more than 22,000 dengue cases and 45 deaths in the 2010 epidemic [9]. Although most dengue outbreaks in Laos have occurred in urban areas, an ecological survey of *Aedes* mosquitoes conducted in a central province of Laos suggested that dengue might be more prevalent in rural than urban areas and that peri-urban areas may be the hotspot for dengue infection [10]. Indeed, dengue was the leading cause of non-malaria febrile illness among patients seen at a rural Salavan Province in southern Laos between 2009–2010 [Mayxay et al., The causes of non-malaria fever in Laos - evidence to inform empirical treatment of fever, submitted].

Despite its severity, dengue is a preventable disease [1]. The only method to prevent the transmission of dengue virus is to control the vector mosquito breeding sites. Good knowledge, attitudes and practices (KAP) among the public are required to successfully prevent or minimize dengue outbreaks. However, very little is known about the public’s KAP on dengue and dengue prevention in Laos. Only one KAP study regarding dengue was conducted in Pakse City of southern Champasack province of Laos in 2009. Out of 230 interviewees, 93.5% knew that dengue was transmitted by mosquitoes and 93.9% recognized water containers as breeding sites for these mosquitoes. The most commonly named symptom was fever, which was named by 75.2% of the participants. However, the participants in this study were comprised of people who had been diagnosed with dengue in the past two years by both symptoms and laboratory tests from the hospital – the results were not able to be generalized to the whole community in Pakse [5].

Understanding the KAP of the general community on dengue and prevention will provide valuable information for effective strategic planning and engaging the public in dengue control. This paper reports on a survey of KAP on dengue and dengue prevention among the general community of Pak-Ngum District – a peri-urban area of Vientiane Capital of Laos.

## Methods

### Study duration and location

A household survey was conducted in January 2009 in Pak-Ngum District - a peri-urban area of Vientiane Capital of Laos. Ethical clearance for the study was granted by the Ethics Committee of the University of Health Sciences, Ministry of Health, Laos.

Pak-Ngum District is approximately 60 Km south of the center of Vientiane Capital and is inhabited predominantly by rice farmers of the Lao Loom ethnic group (~98%) with a GDP of ~1,039 $US. It is home to 53 villages with 9,403 households and a total population of 50,265. There is one district hospital, 9 health centers and 11 private pharmacies in the district. The climate in Pak-Ngum is tropical with a distinct monsoon season (April - October) and a dry season (November - March). The climate tends to be hot and humid throughout the course of the year. Although the number of dengue cases presented and admitted to Pak-Ngum District Hospital was low in recent years (36 cases in 2009 and 54 in 2012), the 2012 *Aedes* mosquito larvae survey found that 91% of households tested positive for *Aedes* larvae and there were 125 containers with *Aedes* larvae per 100 households.

### Sampling procedure and survey

A two-stage cluster sampling method [11-15] was used to select a sample of participants to represent the general community of Pak-Ngum. First, 30 clusters/villages were randomly selected from 40 accessible villages using probability proportionate to size cluster sampling (there were 13 additional villages in this district which could not be accessed). Once the clusters were selected, we used simple random sampling from village household lists to identify 8 households to survey for each cluster. We calculated a desired sample size of 240 households (8 households from each of the 30 clusters). This was based on a 95% confidence level, a ± 10% margin for error, a response distribution of 50% (which requires the largest sample size), and allowance of 10% for non-response and a conservatively estimated design effect of two.

We sought to interview the adult (>18 years of age) head of each of the selected households. After written consent was obtained, the head of each household was face-to-face interviewed by two local interviewers (ST and KP) using an interviewer-administered questionnaire in the local language.

The questionnaire comprised questions on knowledge, attitudes, practices related to dengue and dengue prevention. After completing the questionnaire, the interviewer made an observation of the area around the participant’s dwelling to assess it with regard to dengue prevention. The questions in the questionnaire were determined by reviewing previous KAP studies assessing the levels of knowledge, attitudes and practices regarding dengue. A KAP questionnaire used in Swaziland to test the public’s knowledge on malaria was also used as a template for beginning to build our own questionnaire [16]. The questionnaire was first translated into Lao and then back translated into English to make sure the first translation was accurate. We piloted the questionnaire on students at the University of Health Sciences in Vientiane, Laos.

### Data analysis

Data were entered into a Microsoft Excel spreadsheet and then transferred to Stata version 13.0 for analysis using survey commands to adjust for the two-stage cluster sampling design. Descriptive statistical analysis was used to provide estimates of population proportions with their respective 95% confidence intervals. Cross tabulations were performed with *Chi-square* or *Fisher’s Exact* tests to assess statistical significance (α = 0.05).

## Results

Of the 240 households randomly selected to be surveyed, 231 (96.2%) gave consent and participated in the study. Nine households were not surveyed because no one was at home during the survey despite the third attempt of visit. The socio-demographic details of the informants are shown in Table 1.

**Table 1:** Socio-demographic characteristics of the participants in the survey (n = 231)

**Variable**	**n**	**%**
Gender		
Male	77	33.5
Female	153	66.5
Age (years):		
18 – 24	9	3.9
25 – 34	52	22.6
35 – 44	74	32.2
45 – 54	65	28.3
55 – 64	22	9.6
65+	8	3.5
Education (years):		
0	18	7.9
1 – 6	116	50.7
7 – 12	92	40.2
12+	3	1.3
Occupation		
Farmer	192	83.1
Merchant	16	6.9
Housewife	8	3.5
Labourer	8	3.5
Gardener	4	1.7
Civil servant	1	0.4
Government worker	1	0.4
Unemployed	1	0.4
Religion (Buddhist)	231	100
Previous family experience with dengue (yes)	38	16.4

### Dengue knowledge of and dengue information source received by the respondents

Of all participants, 225 (97.4%, 95% CI = 95.0 - 99.8) had heard of dengue. Most participants could recognize fever as a symptom of dengue and a considerable proportion recognized headache and muscle pain but fewer could name bleeding and skin rash as signs of dengue infection (Table 2). We found that 94.1% (n = 209, 95% CI = 89.2-99.1) knew that mosquitoes were the transmitting vectors, and 93.3% (n = 195) of these knew that *Aedes aegypti* were the specific mosquito that transmitted dengue. Almost all participants [n = 212, (94.2%, 95% CI = 90.6-97.8)] recognized outdoor containers (tyres, river/ponds) as breeding sites for *Aedes* mosquito, but less than 10% recognized that indoor water containers (forest areas, sewage, drains, dirty water, and stagnant water) were also potential breeding sites. A small proportion incorrectly believed areas with running water could be breeding sites for *Aedes* mosquitoes. In addition, 63.6% (n = 143, 95% CI = 51.1-76.0) correctly reported that *Aedes* mosquitoes bite most frequently at sunrise and 93.3% of them (n = 210, 95% CI = 89.2-97.5) correctly identified that rainy season was when *Aedes* mosquitoes were most prevalent.

**Table 2:** Signs and symptoms of dengue recognized by the respondents in the survey (n = 231)

**Symptoms/signs**	**n**	**% (95% C.I.)**
Fever	182	80.9% (71.4 - 90.4)
Headache	103	45.8% (30.9 - 60.7)
Muscle pain	70	31.1% (20.9 - 41.2)
Bleeding	57	25.3% (13.4 - 37.3)
Skin rash	33	14.7% (8.1 - 21.2)
Nausea	16	7.1% (2.5 - 11.7)
Don’t know	32	14.2% (6.9 - 21.5)

*NB*: Multiple response options.

Two-thirds (67.4%, n = 151, 95% CI = 59.3-75.5) of participants knew that dengue and malaria were different diseases, the rest either thought they were the same disease (22.8%, n = 51, 95% CI = 13.9-31.7), or reported that they did not know if they were different (9.8%, n = 22, 95% CI = 5.2-14.5). The majority of interviewees (90.2%, n = 202, 95% CI = 84.5-95.9) knew that malaria was transmitted by a mosquito but there was some confusion between the mosquito vectors for malaria and dengue. Only 28.0% (n = 63, 95% CI = 16.1-39.8) could name the *Anopheles* mosquito as the vector for malaria, while 31.6% (n = 71) incorrectly believed that *Aedes* mosquitoes transmit malaria.

Respondents were asked where they had heard about dengue and where they hoped to get more information. The results are presented in Table 3. The majority [n = 209 (92.9%), 95% CI = 88.7-97.1] of the interviewees did not believe that they had enough information on dengue. Forty-seven percent of them wanted more information on control and prevention methods, and 25.3% wanted to know more about signs and symptoms of dengue.

**Table 3:** Sources of information about dengue received by the respondent in the survey (n = 231)

**Variable**	**n**	**% (95% C.I.)**
Where participants have heard about dengue:		
Television	128	56.9 (46.9 – 66.8)
Friends	125	55.6 (45.0 – 66.1)
Health facilities	112	49.8 (37.9 – 61.6)
Community meetings	91	40.4 (28.0 – 52.9)
Health care workers	83	36.9 (24.5 – 49.3)
Radio	46	20.4 (13.3 – 27.6)
Family	40	17.8 (8.9 – 26.6)
Temple/Church	27	12.0 (6.1 – 17.9)
Poster/pamphlets	5	2.2 (0 – 5.2)
Newspaper	2	0.9 (0 – 2.2)
School	1	0.4 (0 – 1.3)
Where participants hope to hear about dengue:		
Television	114	50.7 (39.0 – 62.3)
Community meetings	107	47.6 (36.9 – 58.1)
Health care workers	94	41.8 (28.6 – 54.9)
Health facilities	78	34.7 (22.9 – 46.3)
Friends	73	32.4 (17.5 – 47.4)
Radio	54	24.0 (15.8 – 32.2)
Temple/Church	22	9.8 (4.1 – 15.4)
Family	22	9.8 (1.4 – 18.1)
Poster/pamphlets	21	9.3 (3.7 – 14.9)
School	2	0.9 (0 – 2.2)
Newspapers	1	0.4 (0 – 1.4)

NB: Multiple response options.

### Attitudes of the respondents on dengue

Ninety-six percent (n = 211, 95% CI = 92.0-99.9) of participants believed that dengue was fatal, but only 28.0% (n = 216, 95% CI = 17.7-38.3) would seek treatment within the first 24 hours. All respondents said they would seek treatment at some stages at a health facility. Ninety-four percent (n = 211, 95% CI = 88.2-99.3) of them believed that dengue was preventable. More than half of these participants (64.9%, n = 146) considered themselves at least partly responsible for dengue prevention, leaving approximately one-third believing that they were not in any way responsible for dengue prevention. Thirty-nine percent (n = 88) mentioned that dengue prevention was the responsibility of health care workers.

### Dengue prevention practice by the respondents

When the participants were asked on what methods they used to protect themselves from *Aedes* mosquito bites, almost all said they would use mosquito nets (Table 4). Most interviewees recognized that water containers were a major breeding site for *Aedes* mosquitoes. Approximately half of the participants reported that they cover their water containers and just less than half reported that they clean water containers to prevent breeding. One quarter reported that they treat the water in water containers. From observation of the respondents’ houses there were signs that participants weren’t taking measures to eradicate *Aedes* breeding sites. There were many yards containing rubbish, water jars and tanks, as well as discarded tyres. Despite 50.7% of participants reporting that they covered their water containers, only 21.5% of water jars and 10.2% of water tanks outside the participants’ dwelling were covered on observation (Table 5).

**Table 4:** Dengue prevention practices reported by the respondents in the survey (n = 231)

**Variable**	**n**	**% (95% C.I.)**
Mosquito bite prevention practices:		
Mosquito nets	223	99.1 (97.8 - 100)
Mosquito coils, mats and liquid vapourisers	94	41.8 (33.3 - 50.3)
Indoor insecticide spraying	80	35.6 (24.5 - 46.6)
Fans	23	10.2 (5.9 - 14.5)
Clothing	13	5.8 (1.6 – 10.0)
Personal mosquito repellents	8	3.6 (1.0 - 6.2)
Window and door screens	1	0.4 (0–1.4)
Methods to eradicate *Aedes* breeding sites:		
Covering water containers	114	50.7 (37.3 - 64.0)
Cleaning water containers regularly	105	46.7 (32.7 - 60.7)
Treated water in water containers	60	26.7 (15.8 - 37.5)
Not store water	52	23.1 (13.3 - 32.9)
Cut down vegetation around the home	43	19.1 (10.5 - 27.7)
Dispose old tyres	25	11.1 (4.1 - 18.1)

*NB*: Multiple response options.

**Table 5:** Dengue prevention practices by the respondents by observing the participants’yards in the survey (n = 231)

	**Yards containing objects**	**Objects filled with water**	**Water collection points that were covered**
	**n (%) [95% C.I.]**	**n (%) [95% C.I.]**	**n (%) [95% C.I.]**
Discarded tyres	63 (27.3%)	7 (11.1%)	
	[13.8 - 40.7]	[1.1 - 21.1]	Not applicable
Cans	46 (19.9%)	2 (4.5%)	
	[10.2 - 29.7]	[0–11.3]	Not applicable
Plastic bottles	47 (20.3%)	0 (0%)	
	[8.6 - 32.1]	[0.00-0.00]	Not applicable
Coconut shells	38 (16.4%)	1 (2.6%)	
	[9.2 - 23.7]	[0–8.2]	Not applicable
Flower vases	9 (3.9%)	2 (22.2%)	
	[0.5 - 7.3]	[0–76.9]	Not applicable
Holes in ground	106 (45.9%)	20 (18.7%)	
	[31.9 - 59.9]	[6.3 - 31.0]	Not applicable
Window/door screens	11 (4.8%)		Not applicable
	[1.1 - 8.4]	Not applicable	
Water jars	190 (82.2%)		41 (21.5%)
	[74.2 - 90.3]	Not applicable	[12.5 - 30.4]
Water tanks	118 (51.1%)		12 (10.2%)
	[38.9 - 63.2]	Not applicable	[2.3 - 18.1]

### Comparison between the groups or variables

There were no statistical differences across gender, education, age, and previous experience with dengue infection between: (1) the respondents who were able to list 0, 1–2, and 3 or more symptoms/signs of dengue; (2) the participants who would seek treatment within 24 hours, 2–3 days, and after 4 or more days if they suspected they had dengue; and (3) the interviewees who reported to use 1, 2, and 3 or more methods to protect themselves from mosquito bites.

If suspected of having dengue, 65.6% of the respondents who were not able to name any dengue signs/symptoms would seek treatment within 24 hours compared to those who were able to list 1–2 symptoms/signs (31.0%), and those who managed to list 3 or more symptoms/signs (15.0%), (p < .01). Seventy-nine percent of the participants who could only name one *Aedes aegypti* breeding site held themselves solely responsible for dengue prevention as compared to 37.0% of those who could name 2, and 37.5% of those who were able to list 3 or more breeding sites (p < .01). These results suggest that attitudes regarding dengue care and prevention are negatively correlated with knowledge.

Those respondents with better knowledge reported better practices. The proportion of respondents who reported using three or more methods to protect themselves from mosquito bites was significantly higher in the group who were able to name three or more symptoms/signs of dengue (40.2%) compared to the group who could name only 1–2 (18.8%) and the group who did not know any dengue symptoms/signs (9.4%), (p < .01). Forty-four percent of the interviewees who named 3 or more *Aedes aegypti* breeding sites reported using more methods (3 or more) to eradicate *Aedes aegypti* breeding sites when compared with those who named 2 breeding sites (25.3%), and those who named one breeding site (5.6%), (p < .01). A higher percentage of participants with less knowledge of breeding sites (one and two) had uncovered water jars in their yard (90.1% and 76.1%, respectively) when compared to those with more knowledge of breeding sites (3 or more – 64.4%), (p < .01).

There was no significant association between treatment behavior and mosquito bite protection practices. However, there was a significantly negative relationship between good attitudes (people believe they are personally at least partly responsible for dengue prevention) and a higher number of protection methods: the proportion of the participants who reported that they used 3 or more protection methods was significantly lower in the group with good attitudes (13.9%) compared with the group with negative attitudes (who does not believe they are responsible for dengue prevention) (29.8%), (p < .01). There was also a statistically negative association between good attitudes and incorrect observed practices (uncovered water jars in yard): the percentage of the interviewees with good attitudes (88.7%) was statistically higher in the group with observed uncovered water jars in yard when compared with the group with observed covered water jars in yard (11.3%) (p < .01).

## Discussion

We conducted a survey to assess the dengue knowledge, attitudes and practices of the Lao villagers in a peri-urban area of Vientiane, Laos and found that although almost all of the participants heard of dengue, the depth of their knowledge on dengue was still lacking. For example, approximately one third of interviewees failed to differentiate between malaria and dengue and incorrectly believed that *Aedes* mosquito transmits malaria; many could not correctly report that *Aedes* mosquitoes bite most frequently at sunrise and sunset and few recognized that indoor water containers could be *Aedes* mosquito breeding sites. A study in a semi-urban town of Malaysia also found that although 95% of the study participants had heard about dengue, their knowledge on dengue transmission and control remained insufficient [17]. Another pilot study among people visiting tertiary care hospitals in Karachi, Pakistan showed that although 90% of the interviewees had heard of dengue, only 38.5% of them were found to have sufficient knowledge about dengue [18]. Another survey among adults of high and low socio-economic groups in Pakistan demonstrated that only 35% of the study sample had adequate knowledge about dengue fever and its vector [19]. Almost all participants in our study appear to want more information about dengue as they believed that they did not have enough dengue information - this is a very encouraging opportunity to improve the depth of their knowledge regarding dengue prevention in the community.

This survey confirmed what was found in a previous study conducted in southern Laos that fever was the most recognized symptom, that most participants recognized mosquitoes as the transmitting vector and knew that water containers were a mosquito breeding sites for dengue [5]. A study in slum areas of metropolitan city of West Bengal demonstrated that approximately 69% of the study subjects recognized fever as the main symptom of dengue but 83% of them were unaware of dengue transmission [20]. The survey in northern Thailand showed that fever (81%) and rash (77%) were the most frequently mentioned symptoms by the study participants [21].

Knowledge of the link between mosquitoes and dengue in the current and previous study [5] in Laos suggests that the mosquito education campaigns across the nation have been effective. However, the respondents in our survey were still confused between dengue and malaria – a finding that was also found in Karachi Pakistan [18].

Lack of personal responsibility was found to be problematic in dengue control in many countries such as Thailand [22], Malaysia [23] and Puerto Rico [24]; and this was also the case in Pakse City of Laos where 22% of the study participants held the government solely accountable [5]. In our current study, although the majority of participants believed that dengue is a severe, yet preventable disease; one-third of them do not think they are at least partly responsible for dengue control.

As found in a study in northern Thailand [21], approximately half of our study participants reported that they cover their water containers in order to minimize the risk of *Aedes* mosquito breeding. However, from observation, many households had uncovered water jars or tanks in their yard, as well as discarded tyres and rubbish that could collect water and provide a possible site for *Aedes* breeding. The reasons why people are not practicing prevention methods is not due to lack of knowledge, but other factors which are as yet unknown.

In this survey, good knowledge was negatively associated with good attitudes amongst our study population. For example, a higher proportion of participants who scored worse in the knowledge section would seek treatment immediately. A possible explanation is apprehension towards unfamiliar symptoms, thus prompting the individual to seek medical advice. We also found that a higher proportion of those with less knowledge would take personal responsibility in dengue prevention.

Dengue knowledge and ways to prevent disease is one of the crucial aspects in the strategy to improve disease prevention, and improving education as a method to promote better usage of prevention practices should be implemented. Insufficient proof to link knowledge as a determinant for improving practice seems to be a recurring theme in much of the literature [25]. Community’s good knowledge on dengue does not often translate into better practices [5,22,25,26]. For example, participants in Kuala Kangsar of Malaysia had good knowledge of dengue prevention practices, yet only half covered their water containers [23]. However, our study found an association between better knowledge and both better self-reported practices and better-observed practices – the finding that was consistent with the report from Malaysia [17]. This was also seen in Taiwan’s successful application of better practices through education and cleanliness programs [27], as well as the great decrease of *Aedes* infestation after education programs in Colima Mexico [28].

Despite the association between better knowledge and better practice, we found that good attitudes did not lead to better self-reported or observed practices. This trend was not what we expected, as improving attitudes have previously been shown in Taiwan to also improve prevention practice use [28]. This shows that having the right attitude does not necessarily mean the participant will translate it into practice, and be motivated to act against dengue infection. This indicates that we need to promote the utilization of protection strategies through encouragement of compliance with prevention practices, which will result in more efficient dengue control. This further highlights that in order to bring about behavioral change we need to implement a multi-factorial approach, which also targets other important facets, such as local compliance and the sustainability of disease control programs.

Limitations of our study include the exclusion of 13 inaccessible villages at the time of data collection which may have introduced selection bias and a possibility that a social acceptability bias could have been introduced as the local researchers conducting the surveys were doctors from the district hospital (participants may have felt pressured to give the answers they felt were socially appropriate). The study would have been improved by the development of scales with a scoring system to measure knowledge, attitudes and practices – this would have added greater weight to measures of association between these three constructs. In addition knowledge and attitudes about dengue could differ by important confounders like age, gender, education, site of survey, social status and other relevant characteristics in the population, which could not be accounted for in this analysis. Further research would benefit from adopting a design that would allow for more detailed multivariate analysis.

## Conclusions

Good dengue prevention demands the involvement of the community. Better information is required that helps guide dengue prevention programmes in their efforts to engage with the community. In summary, in Pak-Ngum district there is a lack of depth of knowledge regarding dengue in the community and observation methods revealed that more needs to be done by community members themselves to prevent the spread of *Aedes* mosquitoes. Fortunately, the majority of the community believes they need more information about dengue. These results will guide future research in this area and help to instruct dengue prevention programs.

## Competing interests

The authors declare that they have no competing interests.

## Authors’ contributions

WC, GA, and MM designed the study including the questionnaire and two-stage cluster sampling methodology. WC, VV, LI, VS, ST, KK and MM piloted and refined the questionnaire in the field and implemented the sampling methodology. MM wrote the first draft of this manuscript. All authors have contributed to the further development of the manuscript. All authors read and approved the final manuscript.

## Pre-publication history

The pre-publication history for this paper can be accessed here:

http://www.biomedcentral.com/1471-2458/13/434/prepub
